# Shining Light on Human Gut Bacteriophages

**DOI:** 10.3389/fcimb.2020.00481

**Published:** 2020-09-10

**Authors:** Emma Guerin, Colin Hill

**Affiliations:** ^1^APC Microbiome Ireland, University College Cork, Cork, Ireland; ^2^School of Microbiology, University College Cork, Cork, Ireland

**Keywords:** bacteriophage, phage-bacteria interactions, gut microbiota, microbiome, virome

## Abstract

The human gut is a complex environment that contains a multitude of microorganisms that are collectively termed the microbiome. Multiple factors have a role to play in driving the composition of human gut bacterial communities either toward homeostasis or the instability that is associated with many disease states. One of the most important forces are likely to be bacteriophages, bacteria-infecting viruses that constitute by far the largest portion of the human gut virome. Despite this, bacteriophages (phages) are the one of the least studied residents of the gut. This is largely due to the challenges associated with studying these difficult to culture entities. Modern high throughput sequencing technologies have played an important role in improving our understanding of the human gut phageome but much of the generated sequencing data remains uncharacterised. Overcoming this requires database-independent bioinformatic pipelines and even those phages that are successfully characterized only provide limited insight into their associated biological properties, and thus most viral sequences have been characterized as “viral dark matter.” Fundamental to understanding the role of phages in shaping the human gut microbiome, and in turn perhaps influencing human health, is how they interact with their bacterial hosts. An essential aspect is the isolation of novel phage-bacteria host pairs by direct isolation through various screening methods, which can transform *in silico* phages into a biological reality. However, this is also beset with multiple challenges including culturing difficulties and the use of traditional methods, such as plaquing, which may bias which phage-host pairs that can be successfully isolated. Phage-bacteria interactions may be influenced by many aspects of complex human gut biology which can be difficult to reproduce under laboratory conditions. Here we discuss some of the main findings associated with the human gut phageome to date including composition, our understanding of phage-host interactions, particularly the observed persistence of virulent phages and their hosts, as well as factors that may influence these highly intricate relationships. We also discuss current methodologies and bottlenecks hindering progression in this field and identify potential steps that may be useful in overcoming these hurdles.

## Introduction

The human microbiome is collectively comprised of trillions of microbial cells originating from all domains of cellular life (Bacteria, Archaea, Eukarya) as well as viruses (Ursell et al., 2012). The microbiome has received significant attention in recent decades, and has been considered as an organ in its own right due to its important role in multiple aspects of human health (O'Hara and Shanahan, 2006; Baquero and Nombela, 2012). A major fraction of these microorganisms reside in the gastrointestinal tract (GIT) where they partake in essential metabolic and physiological processes that impact human biology (Turnbaugh et al., 2007; Sender et al., 2016). Thus, the GIT forms a complex ecosystem where biotic factors and abiotic factors, such as immune components and physiochemical parameters, are intertwined (Mirzaei and Maurice, 2017; Shkoporov and Hill, 2019). A healthy human gut is populated by more than 1,000 bacterial species with up to 90% resolving into two phyla, the Firmicutes and Bacteroidetes (Turnbaugh et al., 2007; Lozupone et al., 2012). These bacterial compositions will generally be stable over years and perhaps even decades (Faith et al., 2013). Perturbations and shifts in the composition, abundance, and diversity of bacterial communities has been associated with multiple disease states (Lozupone et al., 2012). These include inflammatory bowel disease, obesity, diabetes, eczema, cancer and neurological disorders (Giongo et al., 2011; Karlsson et al., 2011; Gkouskou et al., 2014; Goodrich et al., 2014; Casén et al., 2015; Hartstra et al., 2015; Halfvarson et al., 2017; Vogt et al., 2017; Helmink et al., 2019). The driving forces behind these shifts remains a vital question in the field of human gut microbiome research. One of the most understudied aspects of the gut microbiome are viruses of bacteria, bacteriophages (phages). It is well known that phages have important roles in shaping bacterial communities in different environments both by predation or horizontal gene transfer through transduction (Koskella and Meaden, 2013).

Together bacteria and their viruses form the most abundant biological entities in the human gut (Cani, 2018). The virome includes both bacteriophages and eukaryotic viruses; however, phages (phageome) are significantly more abundant (Gregory et al., 2020). Furthermore, recent studies have shown that microbiome composition is driven by phages and not by the eukaryotic viruses which are generally disease or diet associated (Moreno-Gallego et al., 2019). Phage-driven alterations of the bacteriome by direct interactions or potentially via the human immune system have been linked to a number of disease states including inflammatory bowel disease (IBD), diabetes, malnutrition, Parkinson's disease (Norman et al., 2015; Reyes et al., 2015; Manrique et al., 2016; Belleghem et al., 2017; Nguyen et al., 2017; Zhao et al., 2017; Kieser et al., 2018; Ma et al., 2018; Tetz et al., 2018; Clooney et al., 2019; Gogokhia et al., 2019; Zuo et al., 2019). Fecal filtrate transfer (FFT) studies further indicate that the phageome has the potential to shape the microbiome. For example, the administration of a bacteria-free fecal filtrate from healthy donors to patients with *Clostridioides difficile* infection (CDI) was demonstrated to prevent disease recurrence, albeit in only five subjects (Ott et al., 2017). The engraftment of the donor phageome has also been observed following fecal microbiota transplantation (FMT) in CDI patients (Draper et al., 2018).

Modern sequencing technology has been critical in providing insights that would have been impossible with culture-based methods (Shkoporov et al., 2019). However, this methodology is also limited because most viral sequences lack homology with phages in current databases. Up to 86–99% of reads in a dataset can remain uncharacterised and are sometimes referred to as “viral dark matter” (Aggarwala et al., 2017). Many bioinformatic pipelines are database-dependent and this results in the exclusion of the majority of viral sequences from subsequent analyses, potentially resulting in skewed findings (Clooney et al., 2019; Sutton and Hill, 2019; Gregory et al., 2020). In terms of laboratory-based research there are also multiple challenges. Many gut bacteria are extremely difficult to culture, thus making isolation of their associated phages difficult (Browne et al., 2016). Furthermore, many phages are strain specific, mimicking gut conditions *in vitro* is problematic, and host factors such as phase variable expression of host receptors and the existence of multiple phage resistance mechanisms can add another level of complexity (Porter et al., 2020).

This review will discuss our current understanding of the human gut phageome and gut phage-bacteria interactions. We will also discuss the importance of isolating and characterizing novel phages and associated bacterial hosts from the gut while reviewing current bioinformatic and lab-based methodologies as well as their challenges. The ultimate goal is to overcome these hurdles and fully understand how phages shape the microbiome and their role in human health and disease.

## The Human Gut Phageome

The human gut is a densely populated ecosystem within a complex structure with significant variations in physical conditions, abiotic and biotic factors including pH, oxygen, nutrient and water availability, immunoregulators and bile acids (Mirzaei and Maurice, 2017). This creates a gradient of conditions from the mouth to the large intestine resulting in microbial niche specificity (Pereira and Berry, 2017; Bauer et al., 2018). In addition to longitudinal variation, the GIT varies radially due to features such as the lumen, mucosa, villi and crypts (Donaldson et al., 2016; Zhao et al., 2019). Within this complex ecosystem bacteriophages can partake in intricate dynamics with bacteria and can influence microbiome homeostasis.

### Phage Lifecycles

**Figure 1:**
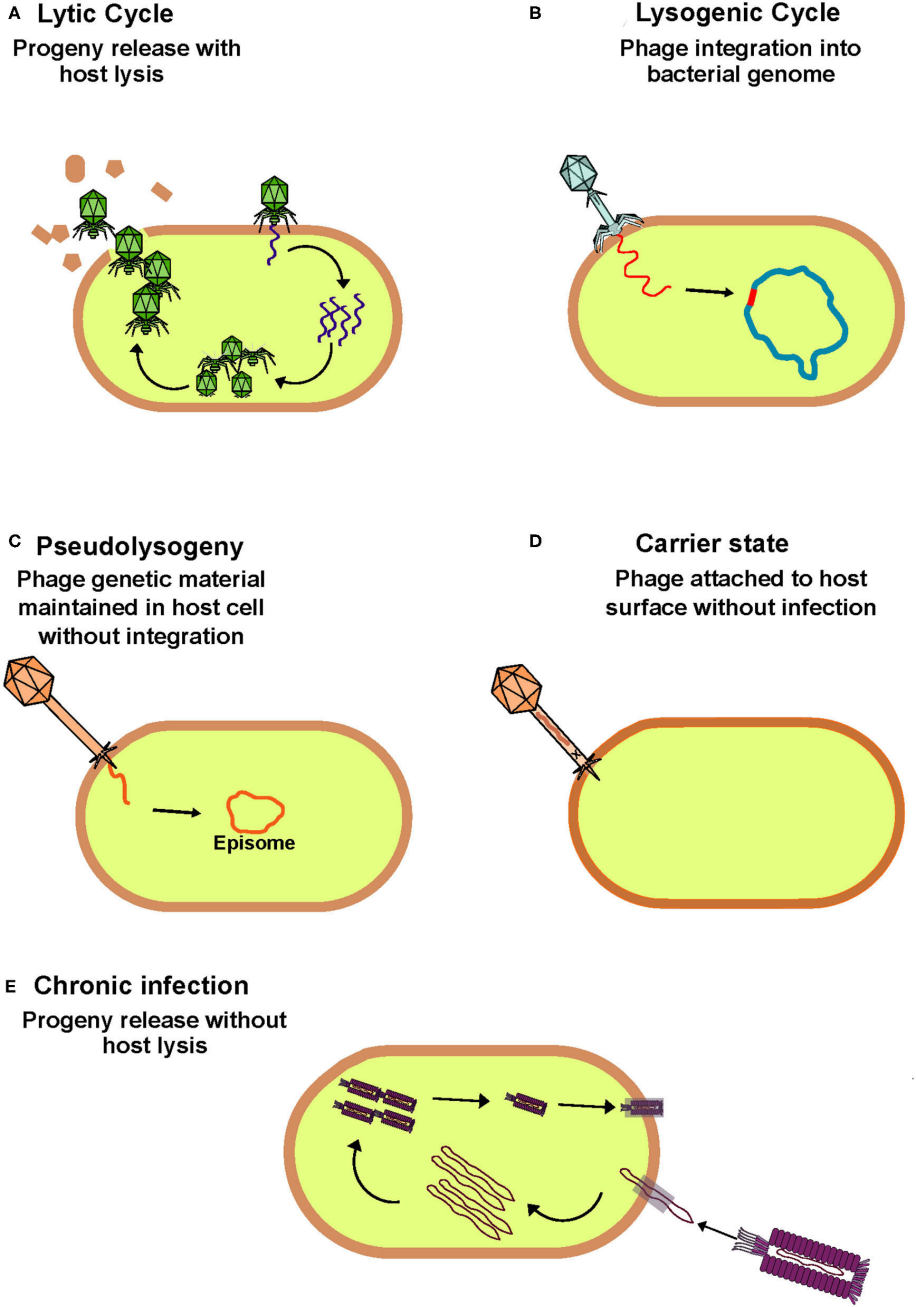
Overview of bacteriophage lifecycles. **(A)** Lytic phages hijack host cell machinery to replicate, assemble and produce progeny which are released from the cytoplasm on host cell lysis to initiation further rounds of infection. This phage lifecycle is thought to be the most prevalent in the healthy human gut. **(B)** Lysogenic phages integrate their genome into the bacterial host genome with which they passively replicate until stress signals trigger their induction and switch to the lytic cycle. **(C)** Phages that follow a pseudolysogenic lifecycle also passively replicate with the host, but their genome remains independent from that of the host and is maintained in the cytoplasm as an episome. **(D)** When phages are in a carrier state they can remain attached to the surface of a non-permissive host without infection. **(E)** During a chronic infection phages produce progeny similarly to that of lytic phages but without host cell lysis.

Traditionally, phages have been classified as lytic/virulent or lysogenic/temperate based on the method of infection they employ when targeting a permissive host (Hobbs and Abedon, 2016). Both life cycles are initiated by adsorption where binding occurs between the phage receptor binding protein and a bacterial cell surface feature which acts as the corresponding receptor. Following this the phage injects its genetic material, which can be either single or double-stranded DNA or RNA, from its capsid into the host cell. It is at this point that the life cycles diverge. Virulent phages hijack host replication machinery to replicate their genome, assemble phage components and produce progeny. This ultimately ends in lysis of the host cell and release of progeny to commence subsequent rounds of host infection. In contrast, lysogenic phages can integrate their genomes into the host chromosome forming a prophage that replicates in tandem with the bacterial genome. Alternative lifecycles such as pseudolysogeny, carrier state and chronic phage infections are less characterized in comparison to the lytic and lysogenic lifecycles (Figure 1). However, the overall prevalence of each of these lifecycles remains poorly understood in the context of the human gut. To avoid ambiguity, when discussing the phageome we generally refer to phages present within bacterial genomes as temperate, while free phage virions are referred to as virus-like particles (VLPs).

Phage lifecycles have an important role in shaping the bacteriome both directly and indirectly. Phages directly drive the bacteriome composition by predation but also through prophage integration or indirectly by horizontal gene transfer (HGT). HGT can confer bacteria with new functionalities and in turn contributes to evolution and the expansion of bacterial diversity in the gut. Temperate phages mediate this transfer of genetic material either by lysogenic conversion or by generalized, specialized, or lateral transduction (Touchon et al., 2017; McInnes et al., 2020). The acquisition of new genes can be favorable to the bacterial host with benefits including improved colonization of the gut, virulence factors, improved stress tolerance, biofilm formation, motility, or immunity in the case of prophages (Obeng et al., 2016). However, the integration of a prophage or new genes can also be detrimental if essential genes are interrupted. If gut commensals acquire genes conducive to virulence, they can evolve to become pathogens, thus, phages can contribute to the spread of virulence and disease development in the human gut. Indeed, prophages or phage genes have been detected in many pathogens. A classic example of this is Shiga-toxin producing *E. coli*. Many non-pathogenic *E. coli* strains reside in the human gut but through the acquisition of phage encoded Shiga toxins, certain strains have evolved into pathogens (Brüssow et al., 2004). Additionally, it is debated that phages carrying antibacterial resistant genes (ARGs) at high abundances, transfer them to bacterial hosts in the gut and fuel the spread of antibiotic resistance. Although, such ARGs have been detected in bacteria it is thought the abundance at which phages carry them is greatly overestimated, largely due to bacterial contamination among viral sequences (Enault et al., 2017; Debroas and Siguret, 2019). It is thought that other mobile genetic elements have a greater role at transferring ARGs among bacteria in the gut than phages (McInnes et al., 2020). The role, if any, that phages play in the dissemination of ARGs in the gut merits further investigation.

### Taxonomy

The International Committee on Taxonomy of Viruses (ICTV) is responsible for the taxonomic classification of prokaryotic viruses which has been traditionally based on virion morphology (Lefkowitz et al., 2018). The best characterized of the phage families are the *Siphoviridae, Podoviridae* and *Myoviridae* that make up the Caudovirales order, all of which are tailed with dsDNA genomes. Members of these phage families have distinct head and tail morphologies: *Siphoviridae* have long non-contractile tails, *Podoviridae* have short non-contractile tails and *Myoviridae* have long contractile tails (Ackermann, 1998). Electron microscopy of human fecal filtrates shows a dominance of these phage morphologies (Hoyles et al., 2014). However, the accuracy of morphology-based taxonomy has been questioned. An interesting argument was put forward by Sutton and Hill regarding the current discrepancies in phage taxonomy, where two phages that shared similar genomic and functional characteristics were classified into different phage families solely based on virion morphology (Sutton and Hill, 2019). This highlights the need for a move toward the use of sequence-based taxonomic classification. Currently, efforts are being made to develop a genome-based taxonomic scheme (Bolduc et al., 2017; Eloe-Fadrosh, 2019; Jang et al., 2019). Indeed, in 2020 ICTV proposed a new 15-rank classification hierarchy of virus taxonomy to accommodate viral genetic diversity (Gorbalenya et al., 2020). This will be important for future virome studies and for the classification of novel phages.

### Composition and Structure

In the human gut, phages are estimated at ~10^10^ g^−1^ while the bacteria they infect equate to ~10^11^ g^−1^ (Shkoporov and Hill, 2019). It has been long postulated that phages greatly outnumber their bacterial hosts at 10:1 virus-to-microbe ratio (VMR) (Bergh et al., 1989; Wommack and Colwell, 2000; Chibani-Chennoufi et al., 2004). VMR has been associated with influencing lifestyle switches of phages (Howard-Varona et al., 2017). With shifts to a high VMR (high phage to low host densities), it was thought that phages would enter a lysogenic life cycle to ensure persistence in the community. However, more recently it has been proposed that with increased bacterial abundances, phages will transition from lysis to lysogeny allowing them to take advantage of the success of their host in “Piggy-Back-The-Winner” dynamics (Knowles et al., 2016). It is important to note however, that this is largely based on marine and aquatic environments and may not necessarily be representative of the human gut. The VMR in the human gut is significantly lower compared to other ecosystems, estimated at ~0.1:1 (Shkoporov and Hill, 2019).

The gut phageome starts to develop in infancy. In the early stages of microbial and phage colonization of the gut, rapid changes in composition occurs (Sharon et al., 2013). A longitudinal study of twenty healthy infant twins observed a shift from a microbiome with high phage-low bacterial diversity at 0 months to a low phage-high bacterial diversity by the time they reached 24 months old (Lim et al., 2015). In comparison to adults, infants have higher bacteriophage diversity but the infant gut phageome is less stable over time than that of an adult. Despite this, both infants and adults have been observed to have high interindividual variation of the phageome (Lim et al., 2016). Although the phageome starts to develop in infanthood, it is still unclear what factors influence the colonization of phages. Proposed influencing factors include diet, genetics, birth mode, vertical transmission, and environment (Lim et al., 2016). The influence of birth mode on infant gut phageome composition is currently debated, however, more recent findings indicate delivery route has less impact on bacteriophage composition compared to bacterial composition (McCann et al., 2018; Maqsood et al., 2019; Siranosian et al., 2020). Interestingly, one study found that as much as 63% of bacterial communities in the infant gut are inherited from the mother compared to 15% of viruses thus suggesting that vertical transmission has minimal influence on phageome composition (Maqsood et al., 2019). This is consistent with the low level of VLPs detected in infant meconium stool but by 1-month high abundances of VLPs are detectable (Liang et al., 2020). It is suggested that soon after birth the neonate gut becomes colonized with pioneer bacteria and prophage induction from these bacteria is the major contributor to the bacteriophage diversity in the early infant gut (Liang et al., 2020). With time there is a shift from temperate phage dominance and high diversity toward decreased richness and abundance by the second year of life with an increase in phages of the *Microviridae* family (Lim et al., 2015). It is now thought that environmental and age-dependent factors likely have an important role in shaping the phageome composition in early life (Maqsood et al., 2019). For example, twin infants have more a shared phageome than that of an older non-twin sibling (Reyes et al., 2015). Furthermore, bacteriophage composition varies between infants that are breastfed or formula fed and mode of feeding also influences eukaryotic virus colonization (Liang et al., 2020). It will be important to further expand on the role of diet and environment in shaping the early life phageome as this may have life-long health implications.

The ratio of virulent and temperate phages in the healthy human adult gut has been a question of interest. The main consensus based on both microscopic and sequence-based virome studies is that the human gut is dominated by temperate dsDNA phages of the Caudovirales order and ssDNA phages of the *Microviridae* family (Reyes et al., 2010; Kim et al., 2011; Minot et al., 2011; Hoyles et al., 2014; Moreno-Gallego et al., 2019). These phages have been described as the core of a healthy human adult phageome with a high occurrence of crAss-like phages (Manrique et al., 2016; Moreno-Gallego et al., 2019). CrAss-like phages form the most abundant phage family in the human gut identified to date with the founder member of the family discovered only in 2014 (Dutilh et al., 2014). These Bacteroidetes-infecting phages showed little to no sequence similarity with phages in pre-2014 viral databases and have the remarkable ability to persist in the gut over extensive periods (Shkoporov et al., 2019). The identified core was found to be highly stable over time which is consistent with reports on crAss-like phages and the slow evolution rates associated with temperate phages compared to virulent phages (Minot et al., 2011; Dutilh et al., 2014; Guerin et al., 2018; Shkoporov et al., 2018a, 2019). Studies have found that 95% of viral genotypes are maintained after 1 year and 80% after 2 years (Reyes et al., 2010; Minot et al., 2013). This was linked to the dominance of temperate phages which have low mutation rates compared to virulent phages, such as *Microviridae*, which have mutation rates as high as 10^−5^ substitutions per day (Minot et al., 2013). Changes in the composition and the richness of the temperate core have also been associated with diseases states such as IBD (Norman et al., 2015). However, the existence of a phage core in the human gut that is shared across individuals has been disputed (Gregory et al., 2020).

A key issue with virome studies and verification of the proposed core phageome is that analytical pipelines tend to focus solely on the known annotated component of viral datasets (~1–14% of sequences generated) and exclude viral dark matter which currently constitutes the majority of sequences (Aggarwala et al., 2017). If only a fraction of the reads is examined this leads to an incomplete and potentially biased analysis. More recent studies have attempted to take this into account by implementing stricter criteria and limiting the use of database-dependent methods. A one-year longitudinal study of the healthy human gut virome of ten individuals implemented a database-independent analysis approach and identified a personal persistent virome (PPV) (Shkoporov et al., 2019). This study clarifies multiple discrepancies in virome studies by showing that temperate phages do not dominate the gut in health as previously thought and confirm that although there is a predominant core of phages in the healthy gut, the idea that a defined set of phages is shared among all individuals cannot be supported which is in agreement with other analyses (Gregory et al., 2020). A total of 22 abundant and temporally stable phage clusters were identified; however, none of these were present in all individuals at one time and the presence/abundance of each cluster was individual specific. These 22 clusters were classified as virulent crAss-like phages, other virulent Caudovirales and *Microviridae*, with only three being identified as temperate. A transiently detected virome (TDV) was also observed but in contrast to the PPV, it was less abundant and less stable but shared to a greater extent across individuals. The TDV is comprised of *Siphoviridae* and phages such as *Inoviridae*. Interestingly, CRISPR-spacer analyses found that the PPV was associated with abundant and stable bacterial genera specifically adapted to a healthy gut environment such as *Bacteroides, Faecalibacterium, Prevotella*, Eubacterium, and *Parabacteroides* whereas more transient genera such as *Escherichia, Akkermansia, Listeria, Clostridium* and *Streptococcus* were associated with the TDV (Shkoporov et al., 2019). In summary, the PPV can be defined as the component of phages in the gut that remain abundant and stable when monitored over 1 year whereas phages that are less abundant and less stable over the same time period are defined as the TDV. These findings also show that the gut is dominated by virulent phages in health as opposed to temperate phages (Reyes et al., 2010; Minot et al., 2011; Moreno-Gallego et al., 2019). If temperate phages are not dominant as previously thought, this begs the question of how virulent phages maintain themselves in the human gut at high levels over extensive periods of time?

With high inter-individual variation across viromes, the identification of viral signals or biomarkers that could differentiate and group individuals based on disease states is challenging. Clooney et al. examined the potential of the virome in distinguishing cohorts through an analysis of healthy and IBD viromes (Norman et al., 2015; Clooney et al., 2019). This was performed using a database-independent method based on the complete virome, i.e., both the known viral sequences and dark matter. Protein-based clustering was performed to identify compositional patterns that potentially separated the two cohorts using vConTACT2, a protein clustering program and the same pipeline implemented by Jang et al. (2019) and Shkoporov et al. (2019). Interestingly, this protocol also revealed a person-specific virulent core of crAss-like phages and *Microviridae* in health which was absent in IBD patients. IBD viromes were found to have an increased abundance of induced temperate phages and this was reflected by a reduction in bacterial alpha-diversity (Clooney et al., 2019). These findings support the observed expansion of specific phage populations in other IBD virome studies (Norman et al., 2015; Duerkop et al., 2018). Duerkop and colleagues proposed that members of the *Spounaviridae* subfamily could serve as a biomarker of colitis (Duerkop et al., 2018). Clooney et al. observed an expansion of temperate *Myoviridae* and *Siphoviridae* (Clooney et al., 2019). As *Spounaviridae* resolve into the *Myoviridae* family, further investigation of this biomarker is merited. In summary, this work shows that loss of, or deviation from, the healthy virulent core to a virome with increased temperate phage induction is indicative of IBD. It would be worthwhile expanding on these findings to identify genus or species-specific changes and apply the same experimental protocol to examine the phageome in other disease states.

## Phage-Host Interactions

The maintenance of a phage in an ecosystem is dependent on its ability to infect a suitable bacterial host. However, bacterial hosts can employ an arsenal of defense mechanisms. This leads to antagonism between the two populations. If selective pressures occur, co-evolution can result in genotypic variants due to mutations at specific sites within both entities, such as in the genes encoding phage tail fibers and bacterial surface receptors. These complex interactions are believed to be an important shaping force of multiple ecosystems. To add another level of complexity there is also evidence that phages interact with the mammalian host, both directly and indirectly, via the immune system (Duerkop and Hooper, 2013; Tetz et al., 2017). For clarity, when discussing phage-host interactions, ‘host' refers solely to the bacterial host unless stated otherwise.

### Host Defense Mechanisms and Phage Counter Defense Strategies

**Figure 2:**
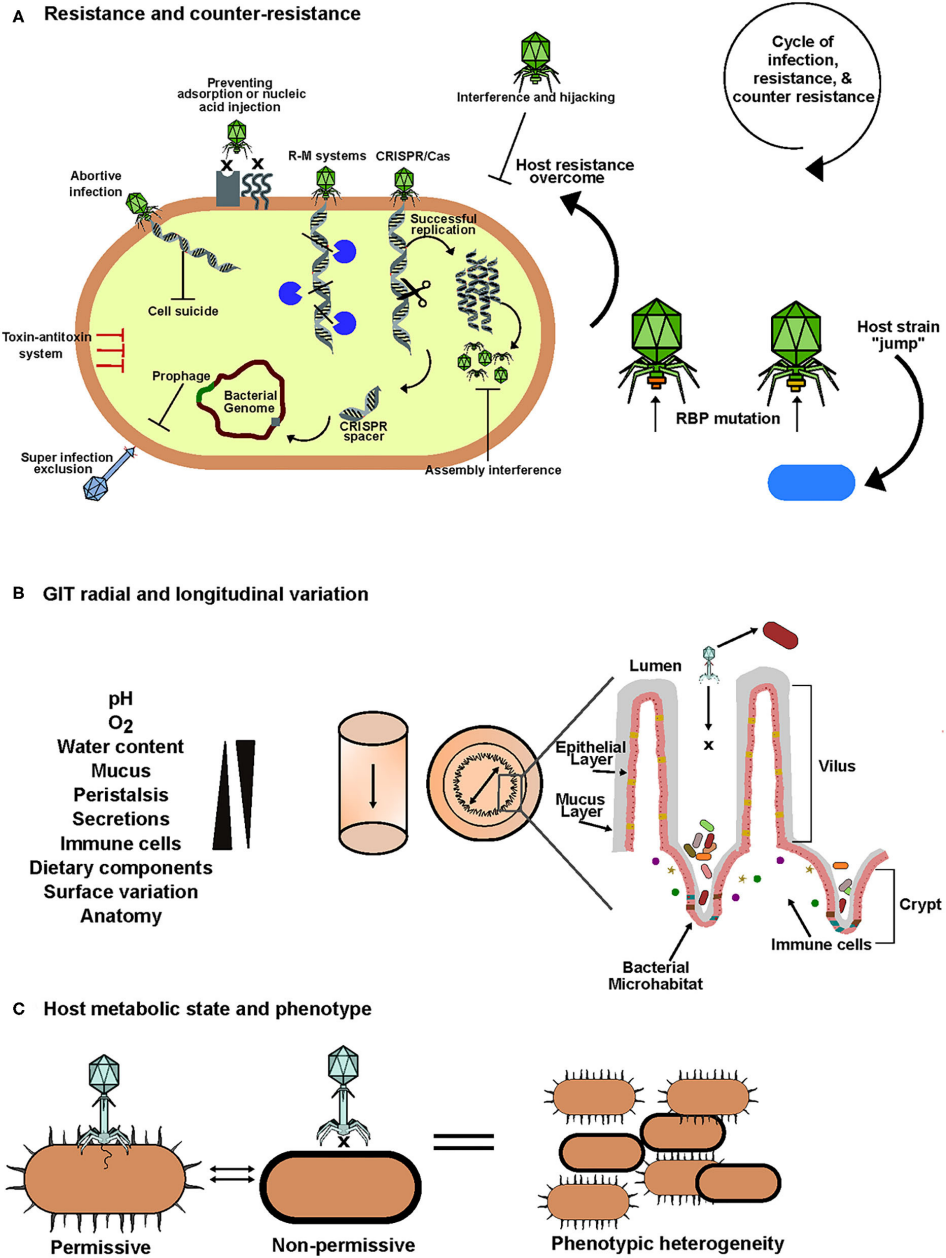
An overview of factors that influence phage-bacteria interactions in the human gut. **(A)** To overcome phage predation, bacteria possess an arsenal of defense mechanisms that target one or more stages of phage infection cycles. In retaliation phages have evolved an array of counter-defense mechanisms. This results in a cycle of infection, resistance and counter-resistance that leads to evolution and diversity in the human gut. **(B)** In the gastrointestinal tract there is significant variation in biotic and abiotic factors both longitudinally and radially. This results in sectional variation in bacterial composition and spatial heterogeneity between phages and their hosts. Anatomical features of the gut such as mucus, crypts and villi create bacterial microhabitats which are inaccessible to phages allowing them to escape predation, gradually seed the lumen and maintain homeostasis. **(C)** Biotic and abiotic factors can also influence the metabolic state of the bacterial host. This may result in transient phenotypic changes mediated by mechanisms such as phase variation allowing adaptation to stress which includes phage infection. These phenotypic changes may inhibit phage infection and can create isogenic populations consisting of phage permissive and non-permissive host variants allowing the co-existence of phage and bacteria.

Both bacterial defense and phage counter-defense strategies have been reviewed extensively (Labrie et al., 2010; Samson et al., 2013; Seed, 2015; Rostøl and Marraffini, 2019). Bacteria can employ an array of defense mechanisms when faced with phage predation. These include preventing phage entry, blocking injection of or elimination of nucleic acids, inhibiting the hijacking of replication machinery and programmed cell death. An overview of these anti-phage mechanisms is provided in (Table 1, Figure 2A).

**Table 1:** A summary of phage defense mechanisms employed by bacteria.

**Site of action**	**Anti-phage mechanism**	**Mode of action**	**References**
Preventing phage entry	Interference with adsorption	Altered expression, mutation or masking of surface features that act as phage receptors and production of polysaccharide capsules	Bertozzi Silva et al., 2016
	Outer membrane vesicles	Vesicles extend from the cell surface, irreversibly bind phages and pinch off to act as a decoy	Manning and Kuehn, 2011; Schwechheimer and Kuehn, 2015; Tzipilevich et al., 2017
	Preventing DNA injection	Alteration of injection site conformation or inner membrane	Cumby et al., 2015
	Superinfection exclusion (Sie) systems	Established lysogens can inhibit secondary infections by other phages by blocking injection of DNA	Cumby et al., 2012
Targeting phage genetic material	Restriction modification systems	Site-specific cleavage of phage DNA	Roberts et al., 2003; Tock and Dryden, 2005
	CRISPR/Cas systems	A form of adaptive immunity, a 20 – 40bp segment of the phage genome is cleaved and integrated into the bacterial genome as a CRISPR-spacer. This allows rapid elimination on repeat infections by spacer sequence directed cleavage of phage DNA	Horvath and Barrangou, 2010; Wiedenheft et al., 2012
	Defense island system associated with restriction–modification (DISARM)	Cleavage of DNA	Ofir et al., 2018
	Bacteriophage exclusion (BREX) system	Prevents DNA replication	Goldfarb et al., 2015
Progeny assembly	Phage-inducible chromosomal islands (PICIs)	Interference with progeny assembly and DNA packaging	Ram et al., 2012; Seed, 2015
Cellular death/dormancy	Abortive infection	The cell shuts itself down to prevent phage replication and release of progeny. This protects sister cells from a similar fate	Dy et al., 2014
	Toxin-anti toxin systems	Target multiple levels of the phage infection cycle. Often leads to cell dormancy or programmed cell death	Rostøl and Marraffini, 2019
	Cyclic-oligonucleotide-based anti-phage signaling system (CBASS)	Mediates genome destruction in infected cells leading to cell death	Cohen et al., 2019; Lau et al., 2020

Despite the efforts of bacteria to ward off phage attack, their predators employ mechanisms to resist. In the case of masked receptors, phages can encode enzymes which degrade capsular polysaccharides (Leiman et al., 2007; Cornelissen et al., 2012). Phages can modify their genomes to avoid restriction modification systems or they can encode anti-CRISPR proteins (Samson et al., 2013; Wiedenheft, 2013). Phages can also mimic host antitoxins to neutralize host produced toxins in host toxin-antitoxin defenses allowing the phage to proceed with propagation (Blower et al., 2012). Interestingly, phage encoded CRISPR-Cas systems have also been detected which allow the phage to hijack host defenses (Seed et al., 2013). When presented with a resistant host with modified receptors, phages can modify their receptor binding protein to continue replication on the same host. For example, lambda phage originally absorbed to its host *Escherichia coli* B, at cognate receptor LamB but with the introduction of four point mutations into the gene encoding its RBP, the phage was able to bind at a new host receptor, OmpF, while maintaining its ability to bind at the original receptor (Chatterjee and Rothenberg, 2012; Meyer et al., 2012). These bacterial adaptations during resistance and phage counter resistance have given rise to co-evolution and arms-race dynamics. This phage driven diversification of bacteria in the gut can have both positive and negative impacts for the human host. These interactions may select for bacteria with phenotypes conducive to disease development or disease resistance (Scanlan, 2017).

### Ecological Models and the GIT

Several ecological models have been developed that describe phage-bacteria interactions in ecosystems. One of the earliest defined ecological models describing these interactions is the classical “kill the winner” model which is derived from the Lotka-Volterra equation (Thingstad, 2000). This model describes the predation of the most abundant bacteria in an ecosystem by their associated phages in a manner that leads to an abrupt reduction in their population. This in turn results in the expansion of another “winner” and a subsequent round of predation leading to fluctuations of the dominant phages and bacteria in an ecosystem (Maslov and Sneppen, 2017). This pattern of predator-prey dynamics has generally not been associated with the GIT, at least not at the genus level (Reyes et al., 2010; Minot et al., 2011; Shkoporov et al., 2019). Furthermore, it does not take lysogenic conversion into account. This is considered in the piggyback-the-winner model which was previously discussed in relation to VMR. Traditionally, this model proposed the lysogenic conversion of phages in a high VMR (high phage: low bacterial densities) to ensure survival until conditions favored reversion to lysis. However, more recently lysogenic conversion was also described in the case of a low VMR (low phage: high bacterial densities) in which phages take advantage of and “piggyback” on host prosperity (Knowles et al., 2016; Silveira and Rohwer, 2016).

Contact rates in the gut can also influence phage-host interactions and perhaps persistence of virulent phages; for example, motility and diffusion can be affected by variations in mucus concentrations. A “continuous time random walk” model was developed supporting this hypothesis (Joiner et al., 2019). Anatomical features in the gut create radial variation and influence the spatial dissemination of phages and their hosts (Figure 2B). An example of such spatial heterogeneity was identified in a study of virulent phage-bacteria coexistence in the gut using a murine model. It was found that the examined phages were in lower abundance in the mucosa while bacterial counts were high and vice versa in the lumen, thus, creating a luminal-mucosal gradient of phages and bacteria (Lourenço et al., 2020). In this example, the mucosa (source) was described as a phage-inaccessible refuge for the bacteria from where they can gradually disseminate into the lumen (sink) and phage predation occurs. These observations suggest that the biogeographical variation of the gut can create source-sink dynamics and spatial distribution that provides certain phage-permissive bacteria with refuges that limit phage predation and thus limits evolutionary selective pressure. As a result, when these bacteria disseminate from their refuge and come in contact with a cognate phage they remain susceptible to infection thus allowing both the bacteria and phage to be maintained. These dynamics can interplay to support the coexistence of virulent phages and susceptible hosts in the gut (Holt, 1985; Lourenço et al., 2020). Interestingly, this is consistent with the idea that populations of intestinal bacteria, particularly commensals, reside in microhabitats such as crypts (Figure 2B). This allows bacteria to form reservoirs and manage the population by re-seeding of the lumen after perturbations (Donaldson et al., 2016). Multiple studies support the idea of spatial refuges for bacteria to avoid or limit phage predation (Schrag and Mittler, 1996; Bruttin et al., 1997; Weiss et al., 2009).

### Other Influencing Factors and Alternative Interactions

The ability of a phage to infect its host is not solely dependent on resistance and counter resistance. The lifestyle and physiological state of the target host is a significant influencing factor on the success of a phage infection. The environmental conditions of the gut have an important role in this due to the impact they can have on the metabolic state and stress levels in bacteria (Figures 2B,C). These conditions include the physical and anatomical variation along the radial and longitudinal axes of the GIT, mucous, bile acids, pH, oxygen, water levels, nutrient levels, peristalsis and dietary components (Koziolek et al., 2015; Vandeputte et al., 2016; Shkoporov and Hill, 2019; Zhao et al., 2019).

The physiological state of a bacterial host can dictate whether it is phage permissive. This can also generate phenotypic heterogeneity within isogenic populations with some showing phage sensitivity while others are resistant (Bull et al., 2014) (Figure 2C). A phenomenon called phase variation is thought to be an important mediator of transient resistance that results in phenotypic variation in response to environmental conditions. Recently, the significance of phase variation in dictating phage-host interactions in the gut has become of interest (Jiang et al., 2019; Turkington et al., 2019; Porter et al., 2020). It occurs by variable expression of gene loci due to hypermutation or methylation resulting in genes being reversibly switched “on or off” (Bayliss, 2009). This is mediated by recombinases, integrases or invertases that act on invertible regions containing promotors of gene loci often associated with expression of surface features such as Sus-like systems, capsule polysaccharides, S-layer and Ton-dependent transporters which can act as phage receptors, outer membrane vesicles, flagella or restriction modification systems (Zaleski et al., 2005; Coward et al., 2006; Nakayama-Imaohji et al., 2009, 2016; Zitomersky et al., 2011; Porter et al., 2020). These phenotypic switches allow the host to adapt to environmental stresses, which are abundant in the human gut, while minimizing the fitness impact on the total population (Moxon et al., 1994). It has been demonstrated that phase variable invertons are more dominant in the human gut compared to other environments (Jiang et al., 2019). Phenotypic heterogeneity is thought to provide bacteria with a form of herd immunity equivalent to that seen among humans. Non-phage permissive host variants limit the viral load and thus the impact on sensitive variants is reduced preventing a drastic reduction in the overall population (Turkington et al., 2019). The use of herd immunity style dynamics to limit phage predation is further supported by the persistent propagation of virulent phages on a microcolony without its elimination (Eriksen et al., 2018).

Interestingly, phages can hijack host quorum sensing to monitor metabolic conditions and population densities, thus granting them the ability to make informed lytic-lysogenic switch decision (Laganenka et al., 2019). Quorum sensing is a phenomenon employed by bacteria to control the expression of specific genes for processes such as virulence and biofilm formation. This occurs through cell-cell communication mediated by bacterial produced extracellular signaling molecules that allow bacteria to monitor population density and make an informed collective decision on whether expression of specific genes is a fitness cost or beneficial to the overall population (Whitehead et al., 2001; Turovskiy et al., 2007; Ng and Bassler, 2009; Papenfort and Bassler, 2016). Bacteria can use this to assess the risk of phage predation and act accordingly. Quorum sensing homologs have also been identified in phage genomes deposited in NCBI databases. More recently, it was shown that *Vibrio cholerae* infecting temperate phage VP882 encodes a QS receptor (VqmA_Phage_) homologous to that of the host allowing it to “listen in” on its target via host produced autoinducer. In turn, the phage can monitor population densities thus allowing an informed lytic/lysogenic lifestyle switch. In high host cell density, lysis occurs resulting in optimal propagation (Silpe and Bassler, 2019). Collectively, the above demonstrates that there are a multitude of factors that influence the interactions between phages and their bacterial hosts. These factors are important for the success of both in the complex gut environment and are summarized in Figure 2.

### Interactions With the Mammalian Host

In addition to phage-bacteria interactions, phages may interact directly or indirectly with the mammalian host, adding another level of complexity when studying the human gut phageome. Ig-like domains have been identified in certain phages which help them adhere to and remain in the outer mucosal layer in the GIT. This led to the proposition of the “bacteriophage adherence to mucus” (BAM) model which describes the idea that such phages protect the mammalian host from bacterial infections (Barr et al., 2013). However, it has also been demonstrated, *in vitro*, that phages can migrate across the mucus layer, cross the gut epithelial barrier or enter systemic circulation either by transcytosis or by peptide-guided transport and subsequently interact directly with eukaryotic cells or cells of both the innate and adaptive immune system (Duerr et al., 2004; Belleghem et al., 2017; Moustafa et al., 2017; Nguyen et al., 2017; Van Belleghem et al., 2019). This was further supported by *in vivo* studies in a germ-free murine model that examined the use of endotoxin-free lytic phages in the alleviation of bacterially driven colon cancer. The administered phages, specifically Caudovirales phage with DNA and not empty capsids, interacted directly with the murine immune system. This was indicated by interferon production (virus-specific immunity) which led to the exacerbation of colon inflammation (Gogokhia et al., 2019). Phages have also been detected in cerebrospinal fluid, possibly due to blood-brain barrier permeability, and have been shown to increase intestinal permeability (Tetz and Tetz, 2018). This can impair gut barrier function leading to leaky gut and components such as bacterial extracellular DNA entering circulation which is associated with inflammation and diseases including cancer (Tetz and Tetz, 2019) Taken together, these findings suggest that certain phages could have also potential roles as human pathogens or contribute to disease development. This has been indeed observed for Parkinson's Disease, type 1 diabetes, type 2 diabetes and gut diseases (Ma et al., 2018; Tetz et al., 2018, 2019; Clooney et al., 2019; Gogokhia et al., 2019) A number of extensive articles discuss these interactions in detail (Tetz and Tetz, 2018; Van Belleghem et al., 2019). Findings thus far suggests that phages can directly influence mammalian health through interaction with eukaryotic cells and indirectly by shaping the gut microbiome altering bacteriome composition or via interactions with the immune system; however, we still have little understanding of the mechanistic details and further studies are required to fully understand how phages can impact the mammalian host.

## Current Methodologies, Challenges, and Potential Solutions

In the past decade there have been significant advances in our understanding of the human gut phageome. Findings to date show that the healthy gut phageome is individual specific and temporally stable comprising a virulent core that is dominated by crAss-like phages, other Caudovirales and *Microviridae*, but which are not shared among all individuals. However, many important questions remain unanswered and our overall understanding of this elusive component of the gut microbiome remains poor in comparison to our understanding of the bacterial fraction which is largely due to methodological challenges. Efforts are ongoing to overcome shortcomings, to develop and standardize protocols, and improve overall reproducibility and comparability.

### Viral Metagenomics, Sequencing and *in silico* Methods

Next generation sequencing technologies, bioinformatic tools and metagenomic studies have provided important insights into the human gut phageome. The majority of these studies have used feces as the sample source of phage genetic material due to the practical difficulties of using other GIT sampling sites (Reyes et al., 2010; Minot et al., 2011; Dutilh et al., 2014; Norman et al., 2015; Guerin et al., 2018; Clooney et al., 2019; Devoto et al., 2019; Shkoporov et al., 2019). In brief, the key protocol steps involved in most viral metagenomics studies includes VLP enrichment, nucleic acid extraction, sequencing library preparation, followed by *in silico* characterization and annotation of generated viral sequences using bioinformatic pipelines. However, due to the lack of universally reproducible methods cross-study comparisons of findings are difficult, and disparities can occur across studies.

The first step in viral metagenomic studies is the removal of non-target contaminants from the biological sample and the enrichment of VLPs to ensure optimal yield and quality of nucleic acids for library preparations (Kleiner et al., 2015). This involves the elimination of prokaryotic and eukaryotic DNA and RNA as well as cellular and dietary debris using physical and chemical process such as homogenisation, filtration, chloroform treatment and enzymatic action (Conceição-Neto et al., 2015). There are multiple phage nucleic acid isolation protocols described and while CsCl gradient centrifugation can yield highly purified samples, this protocol is time consuming, laborious and can introduce bias (Castro-Mejía et al., 2015; Kleiner et al., 2015). It is important to consider that the phageome is comprised of virulent and temperate phages that vary in nucleic acid content (ssDNA, dsDNA, ssRNA, and dsRNA). Developing a protocol to consider all these components is difficult and can affect our overall picture of human gut phage profile. Due to the shared physio-chemical properties among temperate and virulent Caudovirales phages, many techniques have been developed to allow their co-purification. It is important to note that using the incorrect protocol can lead to a biased virome composition (Gregory et al., 2020). The majority of metagenomic studies have focused on enrichment of VLPs followed by extraction of nucleic acids, although many of the protocols promote a biased focus on free-phage and the enrichment of DNA phages (Kleiner et al., 2015).

Protocols may also generate low nucleic acid yields and to overcome this, amplification methods are performed such as multiple-displacement amplification (MDA) (Kim et al., 2011; Marine et al., 2014). However, this leads to the biased amplification of ssDNA viruses such as *Microviridae* by as much as 10-fold which in turn skews phage community composition. This is thought to be the confounding factor behind why certain viral datasets have an extremely high relative abundances of *Microviridae* (Kim et al., 2011; Roux et al., 2016; McCann et al., 2018; d'Humières et al., 2019; Garmaeva et al., 2019; Shkoporov and Hill, 2019; Gregory et al., 2020). Many virome studies interpret findings based on relative abundance and thus results may be skewed where MDA has been implemented (Sutton and Hill, 2019). MDA has also been shown to affect phage diversity and method reproducibility (d'Humières et al., 2019). MDA can introduce GC bias and coverage extremes which can have implications for *de novo* genome assemblies (Chen et al., 2013; Sutton et al., 2019). Having identified the effects of amplification on viral nucleic acids, there is a call to eliminate this step from metagenomic protocols. However, this is challenging due to the variable concentration yields of phage genetic material that can be acquired from samples as a result of differences in the viral load and the strict input requirements for sequencing library preparation (Shkoporov and Hill, 2019). For example, one study found that DNA yields, despite enrichment, could range from 4 to 500 ng of DNA per gram of feces (Shkoporov et al., 2018b). Attempts have been made to improve sequencing library preparations, such as the adaptase-linker amplification method, by excluding the need for MDA with the goal of eliminating downstream issues and providing a more accurate representation of phage composition in a community (Roux et al., 2016).

The elimination of bacterial contamination has also been a hurdle both in terms of viral nucleic acid extraction and *in silico* analyses. Although it is difficult to completely avoid, contamination levels should be limited to minimize impact on the validity of viral sequences deposited in database (Roux et al., 2013). During the enrichment of VLPs and extraction of viral nucleic acids, the choice of filter pore size is important to avoid contaminants. The use of a larger pore size filter such as 0.8 μm is less biased as it allows larger viruses to be retained but this also leads to high levels of bacterial contamination. A pore size of 0.45 is thought to be optimum (Conceição-Neto et al., 2015; Shkoporov et al., 2018b). The consequence of bacterial contamination among viral sequences can impact on conclusions drawn from datasets. This was highlighted in a study that demonstrated the over-representation of antibiotic resistant genes of bacterial origin among phage genomes (Enault et al., 2017). The issue of contamination was further highlight by Zolfo and colleagues by examination of virome sequences from 35 datasets. They detected an abundance of bacterial, archaeal and fungal contaminants irrespective of VLP enrichment technique (Zolfo et al., 2019). Bioinformatic pipeline development and standardization of detection criteria for bacterial contamination is important in ensuring the quality of viral databases and the *de novo* detection of phages. For example, ViromeQC has significant potential in detecting such contaminants. This pipeline was developed to allow stringent quality control of viral sequences using 31 universal bacterial genes in addition to the 16S/18S rRNA genes and 23S/28S rRNA genes (Zolfo et al., 2019). In addition to contamination, Shkoporov et al., also considered the effects of sample storage conditions and operator bias in virome analyses and has proposed the use of an exogenous phage standard to spike fecal samples to allow absolute quantification of viral loads across samples (Shkoporov et al., 2018b). The disparity between relative and absolute quantification in microbiome profiling can be significant (Stämmler et al., 2016; Vandeputte et al., 2017). This issue was recently highlight by Shanahan and Hill. They discussed the issue of microbiome misrepresentation due to relative abundance and how it can mask variations between microbiomes that would be highlighted by absolute abundances (Shanahan and Hill, 2019). Thus, the inclusion of a standard to allow absolute quantification of sequences and viral load should form part of future virome studies to give a more accurate representation of composition. Many protocols in phageome studies have been developed with a particular focus on DNA phages. This results in RNA phages being underrepresented in viral databases and highlights the need for the development of protocols specific to the isolation of these phages (Callanan et al., 2018). The potential array of human gut RNA phages was recently highlighted with the *in silico* discovery of over one thousand near complete RNA phage genomes and over fifteen thousand non-redundant genomes following analysis of 82 publicly available metatranscriptomic datasets, generated from activated sludge and aquatic environments studies, using profile hidden Markov models to detect conserved proteins. Overall this represents a 60-fold increase in identified ssRNA phage genomes (Callanan et al., 2020).

The choice of sequencing platform for virome analysis can influence read length output. Following quality control, reads are assembled into contigs generally by referenced-based or *de novo* assembly methods (Garmaeva et al., 2019). Short read platforms allow deep sequencing with low error rates and require low DNA concentrations. However, *de novo* assembly of short reads is arduous due to the modular nature of phage genomes, strain heterogeneity, high incidences of hypervariable and repeat regions and MDA influenced variations in coverage and GC content (Lima-Mendez et al., 2011; Minot et al., 2012, 2013; Chen et al., 2013; Sutton et al., 2019). This leads to fragmented assemblies and hampers downstream *in silico* analyses (Sutton et al., 2019). It has been suggested that the use of long-read sequencing platforms, such as Oxford Nanopore, which can generate reads that are representative of near complete genomes may overcome the issues associated with short-read sequencing (Somerville et al., 2019; Warwick-Dugdale et al., 2019). The major drawback with this method is that high DNA concentrations are required which, as discussed previously, can be difficult to attain. Therefore, when performing assemblies from short reads, the choice of assembly software and stringent criteria to recruit true viral sequences is essential (Roux et al., 2017; Sutton et al., 2019).

Sequence-based methods have played an important role in providing insights into the human gut phageome. Nevertheless, a pressing issue is the dependency on insufficiently developed viral databases. Most phage sequences in these databases are not annotated due to the lack of homology with known phages. When newly generated sequence reads are aligned to these databases as much as 99% of the reads from a dataset can fail to align to known phage genomes or homologs and thus these reads remain as dark matter (Aggarwala et al., 2017). Therefore, multiple studies have based their findings solely on the identifiable fraction of reads and have excluded the abundant unexplored dark matter. Although these studies have identified cohort variation, it is unknown how the inclusion of viral dark matter would influence and validate the overall findings (Lim et al., 2015; Norman et al., 2015; Monaco et al., 2016; Zuo et al., 2019). To overcome this issue, there has been a move toward the use of database-independent methods, such as open-reference and clustering approaches, to include both the identifiable and unknown fraction of reads in a dataset (Shkoporov et al., 2018b, 2019; Clooney et al., 2019; Moreno-Gallego et al., 2019). Protein-clustering programs, such as vContact2, offer an effective solution and allow the implementation of a whole virome analysis (Bolduc et al., 2017). These work by building a gene-sharing network based on shared protein families across genomes, an approach similar to that employed in the development of the crAss-like phage family taxonomy (Guerin et al., 2018; Yutin et al., 2018; Jang et al., 2019). In addition, this approach overcomes the challenge of detecting cohort variation within datasets due to the high inter-individual variation associated with the human gut phageome (Clooney et al., 2019; Shkoporov et al., 2019; Sutton and Hill, 2019). Also, where annotation is possible, it is important to consider that some phages use alternative genetic codes such as Lak phages and crAss-like phages of specific candidate genera (Guerin et al., 2018; Devoto et al., 2019).

Although the number of phage genomes being generated is increasing rapidly many fail to be taxonomically assigned (Korf et al., 2019). Often the newly deposited phages are *de novo* assembled without *in vitro* characterization and therefore do not necessarily slot into a morphology-based taxonomic scheme (Ackermann, 2012). As a result, there is a significant push toward a genome-based taxonomic classification to allow universal and accurate taxonomic assignment of phages that have not yet been cultured *in vitro* (Meier-Kolthoff and Göker, 2017; Simmonds et al., 2017; Aiewsakun et al., 2018; Eloe-Fadrosh, 2019). This is not without challenges due to the current incompleteness of viral databases, the mosaic structure of phages and the lack of a universal taxonomic marker shared across phages (Lima-Mendez et al., 2011; Shapiro and Putonti, 2018). The application of gene-sharing networks, protein clustering, and whole virome analysis provides possible methodologies to aid the development of such a scheme (Clooney et al., 2019; Jang et al., 2019). This can provide insights into evolution and shared functions across phages (Shkoporov and Hill, 2019). Taken together, this highlights the need for the development, optimisation, and standardization of protocols in many aspects of viral metagenomics and *in silico* tools. Sequence-based analyses have expanded our insights into the composition of the human phageome which would not be possible *in vitro*. Despite this, *in silico* analysis alone can provide little insights into phage-host interactions and biological properties of *de novo* phages as is possible with *in vitro* and *in vivo* methods. Both methodologies in unison will be essential in fully understanding the interactions between phages and the gut microbiome and how these relationships ultimately impact on health.

### *In vitro, ex vivo* and *in vivo* Methods

With recent findings, several questions have arisen regarding the human gut phageome. The observed ability of certain virulent phages to stably co-exist with their host over time remains poorly understood and requires further elucidation (Shkoporov et al., 2018a, 2019). The isolation of dominant gut phages and their hosts, such as the crAss-like phages, which form the virulent core phageome in healthy individuals would provide insights into the mechanisms behind these interactions. CRISPR-spacer analyses have provided some direction for targeting potential hosts (Shkoporov et al., 2019).

Gut bacteria can be difficult to culture as they are often strict anaerobes and the conditions necessary for their growth may be unknown. Metagenomic studies have demonstrated the diversity of bacterial species in the human gut but this is of limited value when an isolate is required *in vitro* (Gill et al., 2006; Turnbaugh et al., 2007; Huttenhower et al., 2012). Until recently, as many as ~80% of the bacteria identified by these studies were uncultured or unculturable (Eckburg et al., 2005). Nevertheless, significant efforts are being made to culture the “unculturable.” As a result, there has been a push toward the isolation and culturing of bacteria using novel techniques with the complement of genome sequencing. Culturing of gut bacteria is not only necessary to improve our understanding of their role in the microbiome, it is also key for investigating phage-host dynamics and the validation and expansion of *in silico* findings. Improvements in culturing methods have played an important role in cultivating bacterial species of the human gut. Media developments have aided the isolation of novel obligate anaerobes from human feces. For example, yeast extract, casitone and fatty acid (YCFA) medium has been shown to support the growth of these bacteria to high levels and can be modified with antibiotics, carbohydrates or other components to select for less abundant species or phenotypes of interest (Duncan et al., 2002; Browne et al., 2016; Das et al., 2018; Forster et al., 2019). Culturomics has also proved to be an important tool in expanding the repertoire of bacterial isolates from the human gut (Bilen et al., 2018). This involves coupling high-throughput culture-dependent and culture-independent methods. Samples from which bacteria are to be isolated are tested in multiple optimized culture conditions and rapid identification of isolates is performed using MALDI-TOF MS and 16S rRNA gene sequencing (Lagier et al., 2012, 2016). In the context of the human gut, culturomics has been successfully applied in the isolation of bacteria from feces, small intestine and colonic samples (Lagier et al., 2016). The potential of this method was extensively reviewed by Lagier and colleagues (Lagier et al., 2018). It is also important to consider screening conditions in relation to phenotypic features. For example, the human gut contains a significant population of spore-forming bacterial species which are troublesome to culture (Abecasis et al., 2013; Rajilić-Stojanović and de Vos, 2014; Browne et al., 2016). Browne and colleagues successfully cultured an array of spore-formers from fecal samples using ethanol shock enrichment to distinguish them from vegetative cells (Riley et al., 1987; Browne et al., 2016). This led to the isolation of 137 bacterial species from six healthy individuals, 90 of which were on the Human Microbiome Project's “most wanted” list of species that had yet to be cultured and sequenced (Browne et al., 2016). This emphasizes that even the most stubborn gut residents can be cultured *in vitro* when the correct conditions are applied; however, identifying these conditions can be difficult.

Fecal samples have been widely used when screening for novel gut phages. Traditional methods of plaque and spot assays are still widely used in this process. This involves screening a phage rich suspension, such as fecal filtrate, from which bacteria and debris have been removed, against a lawn of pure bacterial culture in overlay agar. A successful phage-host pair is indicated by host lysis through spot or plaque formation. These are picked, enriched, and purified on the specific host (Furuse et al., 1978; Kai et al., 1985). Due to the variable abundance of different phages in fecal samples, enrichment is performed to allow phage-host interactions establish and increase titres to detectable levels which is generally indicated by clearing of culture (Salem et al., 2015). This is followed by sequencing of the phage genome. Plaque assays may fail to detect temperate phages unless an induction treatment, such as mitomycin C, is included to induce the phage to a lytic state (Castellazzi et al., 1972). It has also become apparent that not all virulent phages form clear plaques or spots on a suitable host nor do they clear liquid cultures (Porter et al., 2020). This can be due to phenotypic heterogeneity within isogenic populations. Enrichment of phages on intestinal bacteria, either acquired from culture collection or through selective enrichment from feces, followed by shotgun sequencing is a useful approach for screening of novel gut phages. This approach led to the isolation of ΦcrAss001, the first member of the elusive crAss-like phage family to be isolated in pure culture with its host *Bacteroides intestinalis* (Shkoporov et al., 2018a). To achieve this, 20 healthy fecal donors were recruited, fecal filtrates were prepared, pooled and screened against 53 individually cultured bacterial strains in modified YCFA broth under anaerobic conditions over 3 days, with each filtered lysate used to inoculate the subsequent round of enrichment. The enriched lysates were then sequenced to identify expansion on a specific host (Shkoporov et al., 2018a). Chemostats are also convenient for phage-host enrichment from feces. They allow more controlled conditions than can be provided *in vitro* and are useful for examining phage-host interactions (Santiago-Rodriguez et al., 2015a).

Culture-independent methodologies have also been applied in the isolation of novel phage-host pairs. Within biological samples there are constantly a percentage of bacterial cells that either have free virions attached to their surface or contain phages internally either replicating or as prophages. Several protocols take advantage of this to match phages to their hosts. Viral tagging (VT) is a useful tool for making this match. A sample of interest is obtained, and virions are randomly tagged with a generic fluorescent marker that binds to nucleic acids. The tagged phages are then applied to potential bacterial hosts. If interactions occur, fluorescence-activated cell sorting (FACS) can isolate and discriminate between tagged phage-host pairs and free virions and the isolated phage can then be sequenced (Deng et al., 2012, 2014). Single bacterial cells can also be isolated from samples using FACS. This allows complete sequencing of the bacterial genome to be performed and in parallel also capture the genomes of phages which were infecting or attached to the cell. This method of identifying phage-host pairs is called single amplified genomes (SAGs) (Swan et al., 2013; Labonté et al., 2015). It is also possible to directly isolate single uncultured viruses from environmental samples using flow cytometry. This allows for single viral genomics (SVGs) which involves sequencing isolated viruses individually which overcomes certain assembly limitations associated with metagenomics and provides insights into strain variation and genetic diversity (Allen et al., 2011; Martinez-Hernandez et al., 2017). In one case, this method allowed the recovery of the genetic information from 5,000 individual viruses from a marine sample (Martínez et al., 2014). SVGs, however, exclude the host and provides little insight into phage-host pairing or interactions. FACs, although very useful in its applications, is also biased toward the isolation of more abundant viruses (Martinez-Hernandez et al., 2017; Lawrence et al., 2019). VT proves to be a particularly powerful tool for examining phage-host interactions. It has been largely applied with known panels of bacteria but has potential in identifying pairing between both unknown bacteria and novel phages from a specific sample. A workflow incorporating single-cell VT in parallel with metagenomics and SAGs has been developed using human fecal samples which led to the identification of 363 novel phage-host pairs (Džunková et al., 2019).

As mentioned previously studies frequently use feces as the sampling source for examining the virome due to difficulties associated with sampling other sites of the gut and acquiring human biopsies. It has been shown that consistency and transit time of feces can influence bacterial composition and in turn can cause discrepancies in studies, thus highlighting the importance of recording this information (Vandeputte et al., 2016). Feces is the most easily accessible and ethical sample source for examining the gut, although, it is important to consider that phage-host interactions vary radially and longitudinally along the GIT (Maura et al., 2012a; Galtier et al., 2017). Indeed, examination of the virome along different sites of the GIT in non-human primates found that the virome of the large intestine and rectum were similar but distinct from the ileum. These findings indicate that feces provide a good representation of the colon specifically rather than the gut as a whole (Zhao et al., 2019). Another example of sampling site variation was observed in the identification of microbial signatures associated with Crohn's disease in children. Such signatures were observed in the mucosa biopsies but went undetected in assessment of stool samples (Gevers et al., 2014). Therefore, focusing solely on stool samples would have had significant implications for the findings of this study. Nonetheless, feces is a useful proxy for studying the gut phageome but animal models allow for biogeographical variation to be accounted for.

The examination of phages at multiple experimental levels (*in vitro* to *ex vivo* into *in vivo*), the importance of incorporating relevant conditions to experimental models and considering the influence of bacterial metabolic state is essential when examining phage-host interactions and are important factors to consider when developing an experimental pipeline (Lourenço et al., 2018). While *in vitro* studies can be of great importance, they are not necessarily always conducive to realistic conditions. Several studies have demonstrated the usefulness of *in vivo* models in examining the gut phageome (Maura et al., 2012a,b; Reyes et al., 2013; De Sordi et al., 2017; Galtier et al., 2017; Hsu et al., 2019). For example, De Sordi and colleagues examined phage-host strain jumping both *in vitro* and *in vivo* in a conventional murine model, however, the phenomenon was only detected under the latter conditions (De Sordi et al., 2017). This emphasizes the importance of testing under multiple experimental conditions. Murine models or other *in vivo* models can overcome the issue of acquiring samples that would be ethically difficult to access, such as biopsies, and they also allow spatial heterogeneity in the GIT to be considered (De Sordi et al., 2019). Furthermore, overlap between the murine and human gut phageome has been observed. A study that examined the role of intestinal phages in IBD using a murine model of ulcerative colitis, with healthy mice as a control, mapped sequencing reads to contigs assembled from healthy humans and IBD patient fecal viral reads. An overlap of the murine phageome and the human gut phageome was observed in both health and disease states. These findings suggest that murine models are suitable for studying phage-bacteria interactions in the gut (Duerkop et al., 2018).

There are several other techniques that can be incorporated into human gut phageome studies including transcriptome profiling and metatranscriptomics to give insights into gene expression during individual or community phage-host interactions. Few studies have examined this in the context of the human gut. Transcriptome profiling of phage communities in the oral cavity was carried out to compare gene expression in health vs. periodontal disease (Santiago-Rodriguez et al., 2015b). Similar studies should be performed to examine gene expression in a healthy compared to a diseased gut. Additionally, transcriptomics is useful for examining changes in bacterial host gene expression in the presence of a phage (Porter et al., 2020). It can also determine the effect of an experimental model on bacterial host gene expression (Denou et al., 2007). Consequently, this can aid the development of experimental pipelines and avoid inaccurate conclusions. Bioelectronics could be explored as means of examining phage-bacteria interactions in the gut. It has provided many useful biological and biomedical applications (Strakosas et al., 2015; Pitsalidis et al., 2018). With this, it is possible to mimic bacterial and mammalian membranes, called supported lipid bilayers, using bioelectronic tools and membrane biosensors which can detect specific interactions. For example, this method was used to examine membrane interactions with antibiotics to determine antibiotic targets that were discriminatory between bacterial and mammalian membranes (Su et al., 2019). Similar tools were used to monitor *Salmonella typhimurium* infection of epithelial cells (Tria et al., 2014). Bioelectronics could have potential applications for the detailed examination of phage-bacteria interactions.

**Figure 3:**
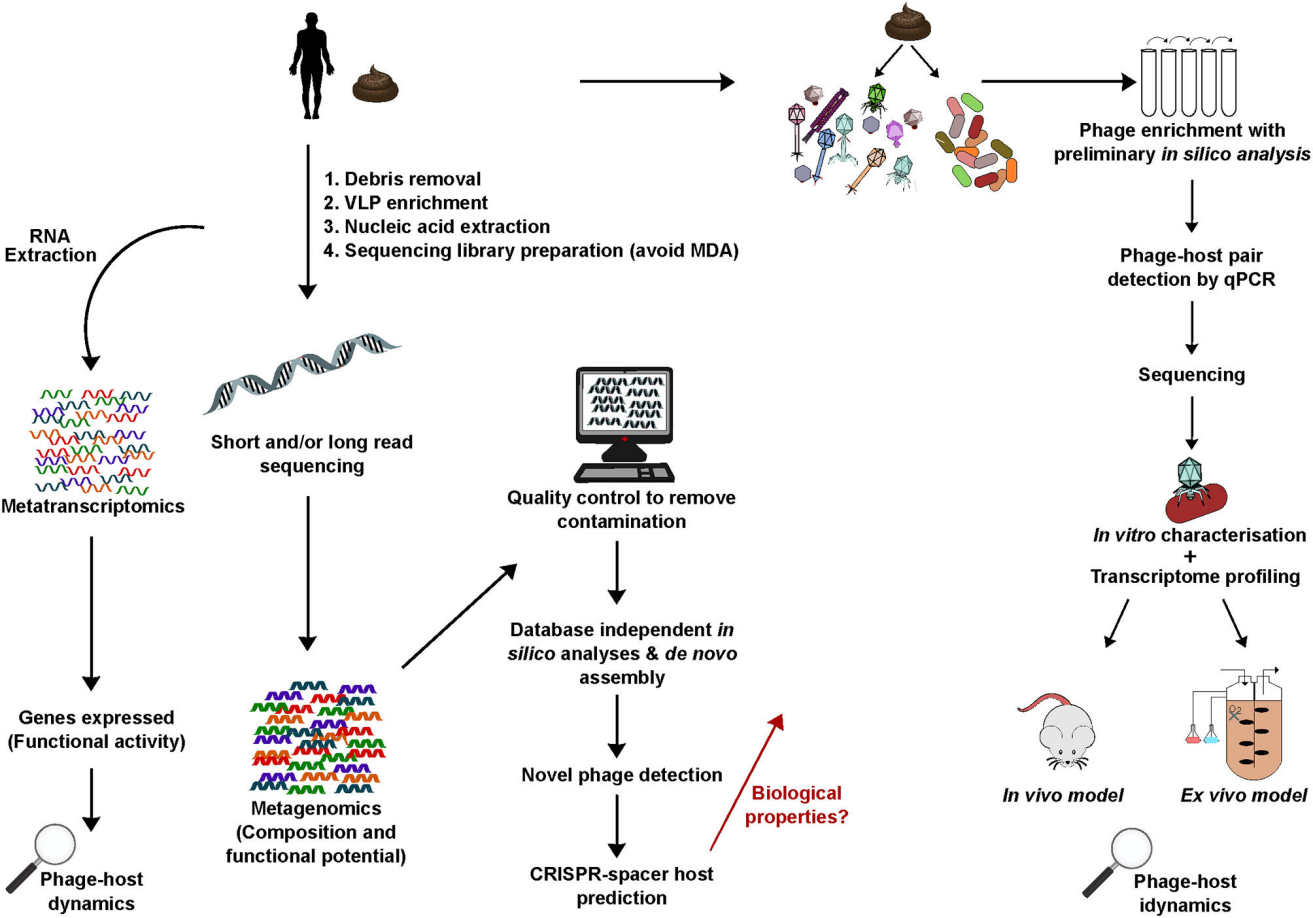
A generic overview of key experimental steps important in studying the human gut phageome: from metagenomics, database-independent whole virome analyses, *in silico* identification of novel phages to isolation and characterization. Linking bioinformatics and lab-based research provides important insights into phageome composition, aids novel phage isolation and characterization of biological properties including interactions with the bacterial and mammalian host.

Methodology can have a greater influence on the virome than the examined disease state (Gregory et al., 2020). The progression in metagenomics is more rapid than the isolation and characterization of novel phage-host pairs from the human gut. To overcome this, both bioinformatics and wet lab work can assist each other to expand on findings. A top-down approach that uses *in silico* findings to guide design of lab-based experiments can allow a more targeted approach. For example, genome sequences of novel phages detected *in silico* following metagenomic sequencing of biological samples can be used to perform host predictions and develop real-time PCR (qRT-PCR) primers to specifically target these phages. This allows the phages to be monitored *in vitro*/*ex vivo* when enriched from the sample of origin on a panel of predicted bacterial hosts thus aiding their isolation in pure culture. When isolation is successful further biological characterisations can be performed while the already available *in silico* data provides information on genome functions and allows relative phages to be identified. Novel phages that have been identified *in silico* need to be isolated and characterized *in vitro*/*in vivo* to understand the mechanisms they employ when interacting with their host, and the health and disease implications that this may have for the human host. Experimental pipelines that bring the above together will be key to this progress (Figure 3).

### Case Study: the crAssphage Family Story; From *de novo* Assembly and *in silico* Characterization to an *in vitro* Reality

**Figure 4:**
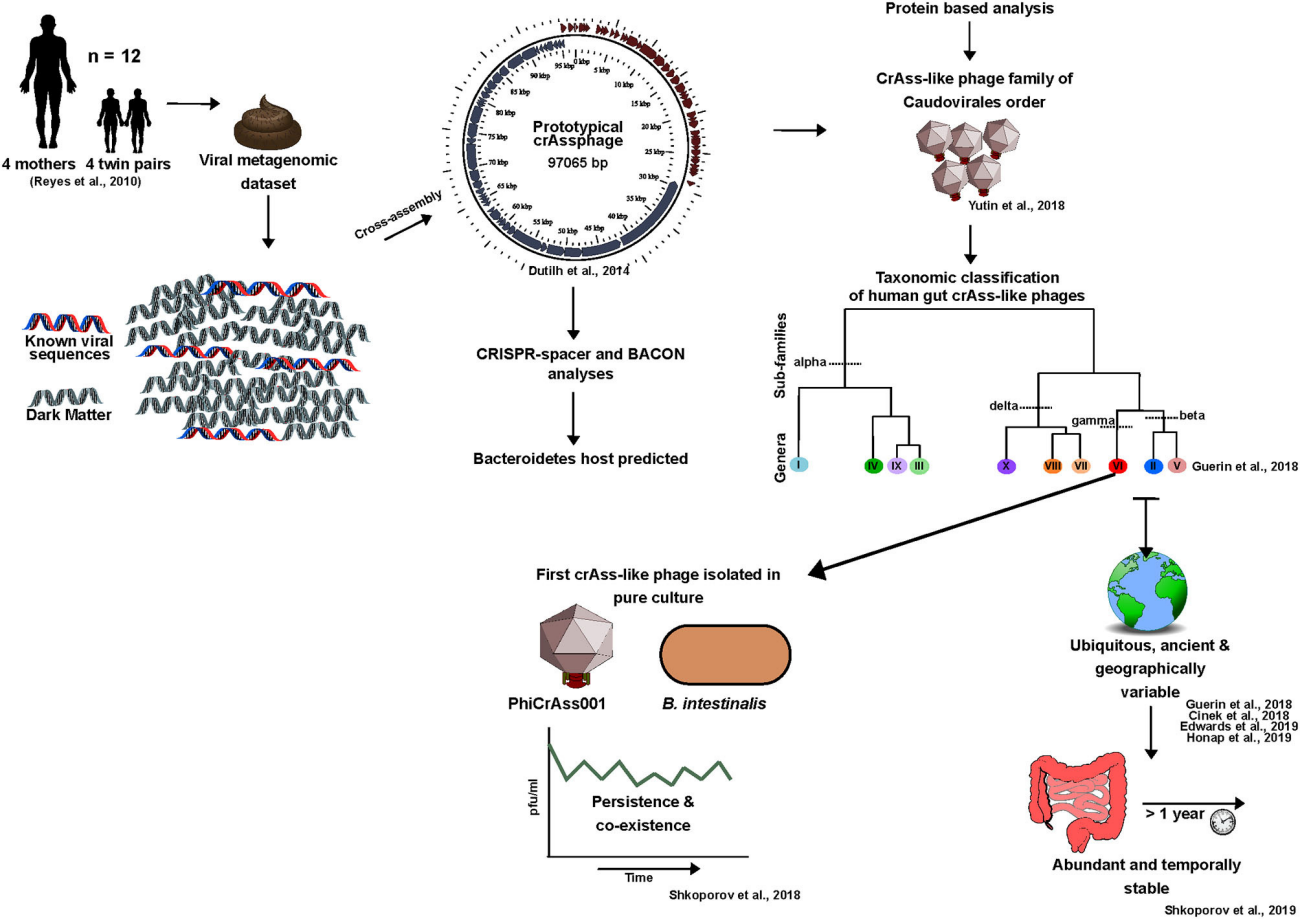
The crAss-like phage family timeline: from *in silico* discovery to an *in vitro* reality (2014-2020).

CrAssphage provides an important example of novel phage discovery using *in silico* methods to overcome the limitations of database-dependency. For years following this discovery crAssphages largely only existed *in silico* due to the challenges faced in achieving isolation on a suitable host. Here, we will discuss the crAssphage timeline from an *in silico* discovery to an *in vitro* reality, and highlight the role that this phage family has played in pushing human gut phage research toward looking beyond the known (Figure 4).

#### Discovery and Importance

Despite one century of phage research, the most abundant phage in the human gut remained undetected until 2014. The majority of its encoded proteins had no homology with phage proteins in public viral databases (Dutilh et al., 2014). In this sense, crAssphage is an ideal representative of the current status of human gut phage research in that it highlights the potential for novel phage discovery within viral dark matter. In the few years since its discovery, this phage has also played an important role in providing essential insights into the human gut phageome.

For clarity, the initial crAssphage detected in 2014 will be referred to as prototypical-crAssphage (p-crAssphage). P-crAssphage was discovered through mining of a previously published human fecal metagenomic dataset (Reyes et al., 2010). Co-occurring contigs were detected based on depth-profile similarities thus ensuring grouped contigs originated from the same phage genome. Using this information, the p-crAssphage genome was *de novo* assembled into a complete circular genome ~97 kbp in size using Cross-Assembly (crAss) software, a reference-independent tool from where the phage received its name (Dutilh et al., 2012, 2014). The novel dsDNA phage was further analyzed against public metagenomes sequenced from human feces in Europe, Korea and the USA and was detected in 73% of the datasets examined. This analysis indicated that the phage was present in ~50% of individuals and was found to be six times more abundant in public metagenomes than all other known phages combined (Dutilh et al., 2014). Despite the abundance at which this phage occurs, it remained undetected prior to 2014 due to the database-dependency of bioinformatic pipelines.

#### Taxonomic Classification and Identification of an Expansive Family

Following studies detected a number of p-crAssphage variants (Liang et al., 2016; Manrique et al., 2016; García-Aljaro et al., 2017). These was further explored in 2018 by Yutin and colleagues through a detailed protein sequence-based analysis of p-crAssphage. This led to the proposition of p-crAssphage as the original member of an expansive family of phages, the crAss-like phage family, which includes the IAS-virus (Oude Munnink et al., 2014; Yutin et al., 2018). These phages were predicted to have a podovirus-like morphology, resolve into the Caudovirales order, and were initially predicted to be predominately temperate (Yutin et al., 2018).

In 2018, a taxonomic classification scheme for crAss-like phages of human gut origin was proposed, with four sub-families (Alphacrassvirinae, Betacrassvirinae, Gammacrassvirinae and Deltacrassvirinae) and ten candidate genera. This was developed using 249 crAss-like phages, 244 of which were *de novo* assembled (Guerin et al., 2018). Members of the same subfamily shared 20–40% orthologous protein similarity and crAss-like phages that clustered into each genus shared >40% protein similarity. Classification was performed using a protein clustering approach that allowed similarity to be identified across the phages despite sharing little to no similarity at the nucleotide level. The developed scheme was further supported by phylogenetic analyses of four conserved genes (capsid, primase, portal protein and terminase) which is consistent with earlier studies (Guerin et al., 2018; Yutin et al., 2018).

#### CrAssphage Host Predictions and *in vitro* Isolation

Through CRISPR-spacer profiling and bacterial co-abundance analysis, it was predicted that crAss-like phages infect bacteria of the *Bacteroides* genus or other members of the Bacteroidetes phylum (Dutilh et al., 2014).

In 2018, the first crAssphage-host pair was isolated in pure culture using a human fecal enrichment protocol and provided some insight into the biological properties of these phages (Shkoporov et al., 2018a). The isolated IAS-like crAssphage, ΦcrAss001, of candidate genus VI, sub-family Betacrassvirinae, infects *Bacteroides intestinalis* thus confirming *in silico* host predictions. Electron micrographs also confirmed the podovirus-like morphology predicted for these phages. The ΦcrAss001 genome is absent of a lysogeny module but intriguingly the phage can co-exist with its host at high titers in co-culture over 3 weeks without significant impact on host growth (Shkoporov et al., 2018a). This is consistent with the observed persistence and stability of crAss-like phages in metagenomics datasets over time (Guerin et al., 2018; Edwards et al., 2019; Shkoporov et al., 2019). This also supports the stable engraftment of these phages in certain individuals for up to a year following FMT (Draper et al., 2018; Siranosian et al., 2020). More recently, the isolation of two crAss-like phages closely related to ΦcrAss001 which infect *Bacteroides thetaiotaomicron* have been reported, DAC15 and DAC17 (Hryckowian et al., 2020). Detailed biological characterization of these phages has yet to be performed but will shine further light on the traits shared across this phage family.

#### Our Current Understanding of CrAss-Like Phages

Thus far we know that crAss-like phages are generally absent from the neonate gut but are acquired in infanthood at low abundances, however, the influence of birth mode on the transmission of these phages has been debated (McCann et al., 2018; Liang et al., 2020; Siranosian et al., 2020). It was initially thought that crAss-like phages were absent from the infant gut, however, now it is believed that they can be vertically transferred from mother to infant (Siranosian et al., 2020). The early colonization of crAss-like phages and their universal ubiquity is not surprising considering the predominance of Bacteroidetes in the healthy human gut from infancy (Rodríguez et al., 2015). These phages have also been detected in the elderly virome (Stockdale et al., 2018). The crAssphage family is globally distributed, with one or more strain being detected in 77% of individuals with geographic clustering (Cinek et al., 2018; Guerin et al., 2018; Edwards et al., 2019). For example, p-crAssphage (candidate genus I), is largely absent from the gut of hunter-gatherer populations and is more abundant among industrialized populations (Honap et al., 2020). CrAss-like phages have not been linked to factors such as disease, age, gender, or body mass index, but a weak link to diet has been detected as well as potential links to ethnicity and geography (Guerin et al., 2018; Edwards et al., 2019; Honap et al., 2020). Diet may be one factor driving the crAssphage family variation observed between Western and more rural non-industrialized populations. The Western diet generally drives a microbiota dominated in *Bacteroides*/*Clostridia*, whereas a non-Western fiber rich diet is associated with high *Prevotella*/low *Bacteroides*. Therefore, this diet driven variation in dominant bacterial species in the gut may also influence crAss-like phage composition (Gorvitovskaia et al., 2016; Guerin et al., 2018).

One of the most intriguing characteristics of crAss-like phages is their ability to persist in the human gut (Guerin et al., 2018; Shkoporov et al., 2018a). With these phages forming a major component of the predominantly virulent personal persistent virome, mechanisms other than lysogeny must be at play to maintain these phages in the human gut over time (Shkoporov et al., 2019). This is supported by the absence of lysogeny genes in the ΦcrAss001 genome and crAss-like prophages going undetected in Bacteroidales genomes deposited in databases such as NCBI RefSeq (Shkoporov et al., 2018a). Although, it should be noted that lysogeny-modules have been predicted for some crAss-like phages detected *in silico* (Yutin et al., 2018). “Royal-family” model dynamics may provide an explanation for the observed stability. If kill-the-winner dynamics occur at a strain or sub-strain level, stability is maintained at the species or genus taxonomic levels over time or it is possible that these phages engage in “piggyback-the-winner” dynamics (Silveira and Rohwer, 2016; Breitbart et al., 2018). The spatial heterogeneity of the gut also provides hosts with phage-inaccessible reservoirs in microhabitats creating source-sink dynamics that reduce selective pressures and allow the coexistence of both (Lourenço et al., 2020). Additionally, the oscillation of bacteria between phage-permissive and non-permissive phenotypes could also have an important role in this persistence (Porter et al., 2020). Overall, it is likely that the persistence of crAss-like phages in the human gut is due to an inter-play of multiple mechanisms.

The isolation of further crAss-like phage representatives will be necessary to identify shared and unique biological properties across this family. The precise mechanisms mediating the stable and persistent colonization of these phages at high levels also needs to be further explored. Ideally, future studies should use *in vivo* models that simulate gut conditions as best as possible. The goal will be to understand how these phages shape our microbiome and influence health. Although we still have a lot to learn about these enigmatic phages, they have played a significant role in highlighting the potential of viral dark matter and in improving our understanding of the human gut phageome both in terms of composition and interactions (Figure 4).

## The Merits of Studying Bacteriophages and Future Prospects

Phages have many potential applications which could be beneficial to humans in addition to forming an important component of the microbiome. The resurging interest in phages largely coincides with the current and projected severity of the antibiotic resistance crises. It is estimated that the inability to treat bacterial diseases due to multi-drug resistance may lead to 10 million additional deaths globally per annum by 2,050 (Sugden et al., 2016). Phage therapy is a potential alternative or aid to antibiotic treatment, however, hurdles associated with this include undesirable immune responses, host resistance development, identifying suitable phage(s), and regulatory issues (Oechslin, 2018; Brüssow, 2019). Several human diseases, such as IBD, have a bacterial component that worsens disease status due to over-stimulation of the immune system (Gevers et al., 2014). Phage-driven selective elimination of pathogens from the gut to shape microbiome composition toward homeostasis is also being examined. The ability of phages to shape the microbiome is supported by outcomes of FFT/FMT studies (Ott et al., 2017; Draper et al., 2018). Phages also produce endolysins which degrade the cell wall of their associated bacterial host and have therapeutic potential as alternatives to antibiotics (Love et al., 2018). While phage therapy is not within the scope of this review, we will mention a few cases of its application. The current status of phage therapy has been extensively reviewed and assessed (Czaplewski et al., 2016; Cisek et al., 2017; Lin et al., 2017; Lourenço et al., 2018; Brüssow, 2019; Hyman, 2019). Phages have also been proposed as alternatives to preservatives or antibiotics in food production and biocontrol (O'Sullivan et al., 2019; Lewis and Hill, 2020; Rabiey et al., 2020). We have seen the antimicrobial capacity of phages in selectively eradicating infection and alleviating diseases with a microbial component. Recently, phages were successfully administered in eliminating cytolytic *E. faecalis* in humanized mice, a pathogen responsible for increased disease severity and mortality in patients with alcoholic hepatitis (Duan et al., 2019). Phage-guided nanoparticles have been used in the eradication of pro-tumoral *Fusobacterium nucleatum* and allowed the site-specific delivery of chemotherapeutic drugs in parallel. This resulted in colorectal cancer tumor reduction in a mouse model with limited impact on healthy tissue (Zheng et al., 2019). This technology was also applied in a piglet model without induction of the immune system or adverse effects (Zheng et al., 2019). However, phage associated immune activation resulting in the exacerbation of symptoms has also be observed (Gogokhia et al., 2019). Remarkably, phage therapy led to the eradication of multi-drug resistant *Klebsiella pneumoniae* by oral and rectal administration of one lytic phage in a human patient without any adverse effects (Corbellino et al., 2019). Phages can also act in synergy with innate immune cells to eliminated pathogens. This was observed in a murine lung model of drug resistant *Pseudomonas aeruginosa* in which phages could only eradicate the pneumonia-causing pathogen in synergy with neutrophils (Roach et al., 2017). These are some examples of the potential of phage therapy in selectively eliminating pathogens. Nonetheless, we need to fully decipher phage dynamics with both the bacterial and human host before we apply phages for therapeutic use. Considering the impending outcome of the antibiotic resistance crisis and the importance of microbiome homeostasis we need to expand our knowledge rapidly.

As we begin to understand phage-host dynamics and how these influence the microbiome, specific phages or phage compositions may be identified as biomarkers of disease or health. For example, whole virome analysis of the IBD and healthy virome identified a shift from a lytic core of a few but abundant clusters of *Microviridae* and crAss-like phages in health to a virome with increased induction of temperate phages such as *Myoviridae* and *Siphoviridae* in the disease state (Clooney et al., 2019). Further development of these findings and the identification of compositional patterns in the context of other diseases states may lead to the use of virome biomarkers in the future. Elucidation of the personal persistent virome will serve as an important window into the gut phageome and provide us with insights into ecology, composition and dynamics including the persistence of virulent phages.

## Conclusions

One of the critical shortcomings of human gut phage research is the incomplete analysis of metagenomic datasets due to the dependency on a poorly curated viral database. Furthermore, little progress has been made in the isolation and characterization of novel gut phage-host pairs. In recent years, efforts are being made to overcome bottlenecks. This requires the development of universal and easily reproducible methods that limit bias during viral enrichment, nucleic acid extraction, and sequencing library preparation. *In silico* analyses need to be performed using database-independent methods that allow for a complete virome analysis using benchmarked criteria. The development of a sequence-based taxonomic scheme is needed to facilitate the rapid expansion of phage sequences as a result of high-throughput sequencing technology. The current contradictions among different gut virome studies need to be rectified and clarified. Overall progression in metagenomic analyses of the gut phageome has been more rapid than the isolation of gut phages which is ultimately the key for expanding our scope of the mechanisms mediating phage-host relationships. Recent findings have provided important insights, but they also highlight how little we know about this important and enigmatic component of our gut.

## Author Contributions

EG and CH drafted and approved this manuscript.

## Conflict of Interest

The authors declare that the research was conducted in the absence of any commercial or financial relationships that could be construed as a potential conflict of interest.
